# MMAP: a cloud computing platform for mining the maximum accuracy of predicting phenotypes from genotypes

**DOI:** 10.1093/bioinformatics/btaa824

**Published:** 2020-11-25

**Authors:** Wei Huang, Ping Zheng, Zhenhai Cui, Zhuo Li, Yifeng Gao, Helong Yu, You Tang, Xiaohui Yuan, Zhiwu Zhang

**Affiliations:** Economic and Management School, Jilin Agricultural Science and Technology University, Jilin, China; Institute of Electrical and Information, Northeast Agricultural University, Harbin, China; College of Life Sciences and Technology, Shenyang Agricultural University, Liaoning, China; Electrical and Information Engineering College, JiLin Agricultural Science and Technology University, Jilin, China; Electrical and Information Engineering College, JiLin Agricultural Science and Technology University, Jilin, China; Information Technology Academy, Jilin Agricultural University, Changchun, China; Electrical and Information Engineering College, JiLin Agricultural Science and Technology University, Jilin, China; Information Technology Academy, Jilin Agricultural University, Changchun, China; Department of Computer Sciences, Wuhan University of Technology, Wuhan, China; Department of Crop and Soil Sciences, Washington State University, Pullman, WA, USA

## Abstract

Accurately predicting phenotypes from genotypes holds great promise to improve health management in humans and animals, and breeding efficiency in animals and plants. Although many prediction methods have been developed, the optimal method differs across datasets due to multiple factors, including species, environments, populations and traits of interest. Studies have demonstrated that the number of genes underlying a trait and its heritability are the two key factors that determine which method fits the trait the best. In many cases, however, these two factors are unknown for the traits of interest. We developed a cloud computing platform for Mining the Maximum Accuracy of Predicting phenotypes from genotypes (MMAP) using unsupervised learning on publicly available real data and simulated data. MMAP provides a user interface to upload input data, manage projects and analyses and download the output results. The platform is free for the public to conduct computations for predicting phenotypes and genetic merit using the best prediction method optimized from many available ones, including Ridge Regression, gBLUP, compressed BLUP, Bayesian LASSO, Bayes A, B, Cpi and many more. Users can also use the platform to conduct data analyses with any methods of their choice. It is expected that extensive usage of MMAP would enrich the training data, which in turn results in continual improvement of the identification of the best method for use with particular traits.

**Availability and implementation:**

The MMAP user manual, tutorials and example datasets are available at http://zzlab.net/MMAP.

**Supplementary information:**

Supplementary data are available at *Bioinformatics* online.

## 1 Introduction

Accurate prediction of phenotypes from genotypes is one of the ultimate goals of genomic research, so that a medical treatment could be optimized to improve human and animal health, and breeding could be revamped to increase animal and plant production. Before a complete identification of genes underlying a particular trait of interest through techniques, such as genome-wide association study (GWAS), genomic prediction or genomic selection (GS), is a practical shortcut that plays a critical role in animal and plant breeding to predict phenotypes from genotypes without knowledge of where those genes are. Many statistical methods and computing tools have been developed to conduct GWAS and GS, including the common methods and tools for both GWAS and GS (Endelman, 2011; Kim *et al.*, 2019; Lipka *et al.*, 2012; Pérez and De Los Campos, 2004; Tang *et al.*, 2016). However, there is a fundamental difference between GWAS and GS. There are minimal interactions between GWAS methods and traits. For example, for some traits, all methods perform the same, either successfully detecting a major gene or failing to detect any association when either sample size or gene effects are too small. For other traits, these methods perform differently. Some methods detect more associations than others. The magnitude of the statistical power varies from trait to trait. However, the orders of methods rarely change. The situation is different for GS. The order of GS methods varies from trait to trait depending on the genetic architecture of the traits (Wang *et al.*, 2018). For polygenic traits, genomic Best Linear Unbiased Prediction (gBLUP) performs better than SUPER BLUP. For Mendelian traits, the opposite is true. For traits with low heritability, compressed BLUP performs better than Bayesian LASSO, and the reverse applies for traits with high heritability (Wang *et al.*, 2018). It is challenging to choose a suitable method for a particular trait. Researchers have to examine a variety of methods before reaching a desirable prediction accuracy. Additional challenges, such as installation, steep learning curves and required computational resources intimidate many biological researchers. There is a critical need to develop a free computing platform that would automatically identify the best method and conduct analyses for users with minimal effort, such as uploading and downloading genotype and phenotype data. Herein, we present a cloud computing platform to solve the problem by Mining the Maximum Accuracy of Predicting phenotypes from genotypes (MMAP).

## 2 Method and implementation

**Fig. 1.**
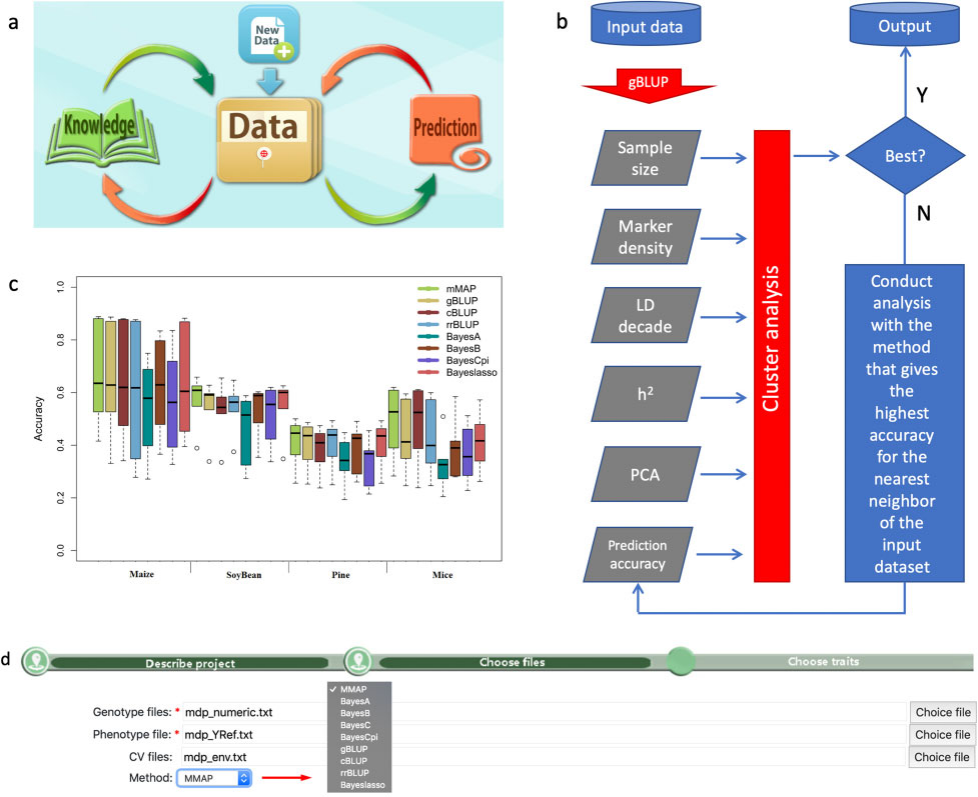
The workflow and performance of MMAP. As a cloud computing platform, MMAP integrate existing knowledge and interactively search for the best GS method for a particular dataset (**a**). The search is based on the characteristics of the input data and IDE initiated with the gBLUP method (**b**). MMAP has the highest average prediction accuracy (**c**) with minimal effort required for uploading phenotypic data, genotypic data and covariable data (**d**)

MMAP is a knowledge-based cloud computing platform that continuously gains knowledge over time during application (Fig. 1a). It currently implements eight GS methods and a mining system to identify the best prediction method for a particular trait (Fig. 1b and Supplementary Fig. S1). The eight GS methods include gBLUP, compressed BLUP, SUPER BLUP, Bayes A, Bayes B, Bayes C, Bayes Cpi and Bayesian LASSO (Fig. 1c). The mining system consists of an existing database and an interactive and dynamic evaluation (IDE) across GS methods and datasets. The current database contains the essential characteristics of over a hundred datasets and their prediction accuracy using these GS methods (Supplementary Tables S1 and S2).

The essential characteristics include sample size, genome size, number of markers, linkage disequilibrium decade, heritability and parameters of principal component analysis. The IDE contains an initial evaluation of prediction accuracy using gBLUP, which was reported to have the highest prediction accuracy on substantial traits, especially the polygenic traits. The essential characteristics and the initial prediction accuracy using gBLUP are used as the input to predict the next methods with two objectives. First, the next method has a high probability of being the best among the implemented GS methods. The other objective is to provide relevant information to find the method that has the highest chance to be the next best GS method. We implemented single trait GS methods and the IDE using the efficient C/C++ programming language and incorporated several highly efficient open-source mathematical operation and optimization libraries. The computation is distributed across multiple nodes on our networked Linux High-Performance Computing cluster.

## 3 Workflow and user interface

MMAP has four tabs to navigate in the platform operations, including User Account, File tab, Project tab and user manual. The File tab navigates to upload input data for phenotypes, genotypes and covariate variables. The Project tab specifies the input data and provides the link to download prediction results (Fig. 1d).

## 4 Results and discussion

The prediction methods implemented in MMAP can be selected specifically to generate identical or similar results depending on methods using random sampling or not (Supplementary Figs S2 and S3). Under automatic mode, MMAP took an average of 2.93 times to find the best method at 91% success, and 96% success at identifying at least one of the top three methods (Supplementary Fig. S4). Among the multiple traits across four species examined, MMAP had the highest average prediction accuracy compared to all implemented GS methods (Fig. 1c and Supplementary Figs S5 and S6).

## 5 Conclusion

MMAP is a cloud computing platform with a user-friendly interface that requires minimal effort to conduct GS without an explicit understanding of a variety of methods and computing tools. Researchers are entirely liberated from software installation, training with steep learning curves and allocating appropriate computing resources with this free platform.

## Supplementary Material

btaa824_Supplementary_Data
