# Supplementary material for: MMAP: a cloud computing platform for mining the maximum accuracy of predicting phenotypes from genotypes
Source: Bioinformatics. 2020 Nov 25;37(9):1324–6. doi: 10.1093/bioinformatics/btaa824 (PMC8189680; doi:10.1093/bioinformatics/btaa824)
Supplement: btaa824_Supplementary_Data [file btaa824_supplementary_data.docx]

**Supplementary**


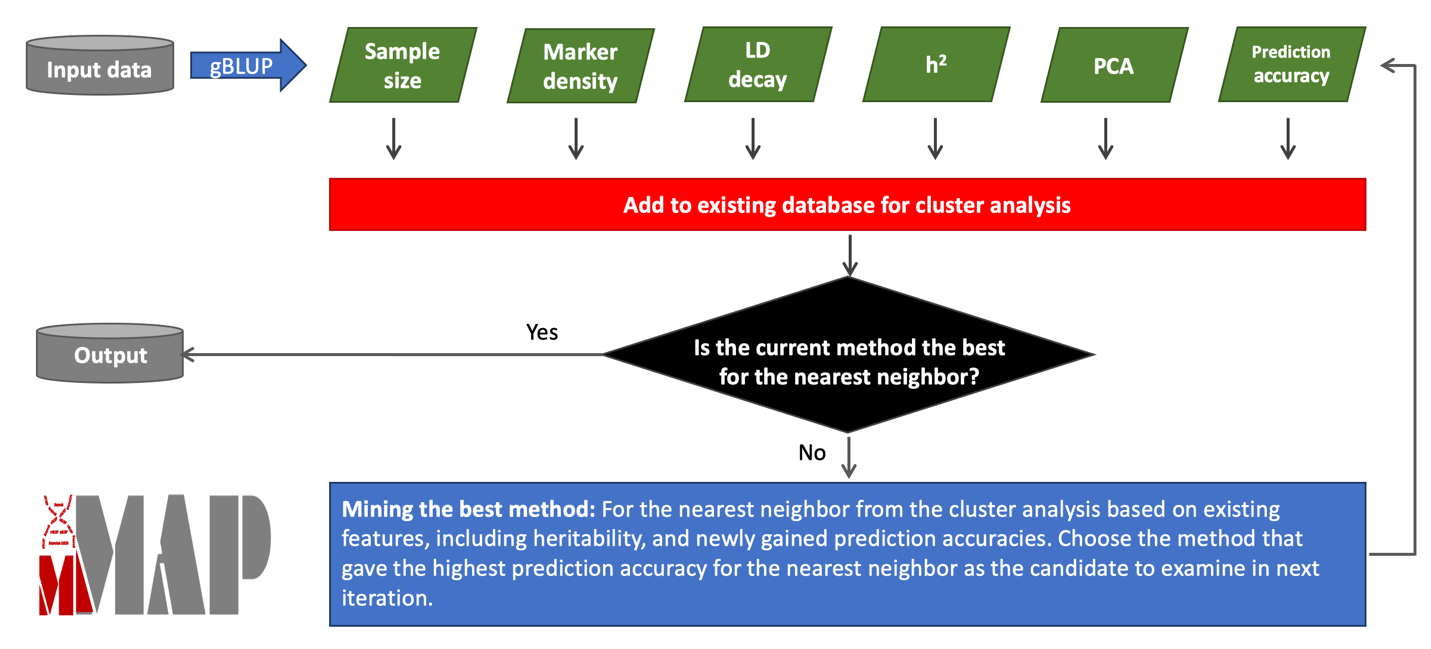


**Figure S1. Procedure of mining the maximum accuracy of prediction of phenotypes from genotypes.** A new input data set is analyzed with genomic Best Linear Unbiased Prediction (gBLUP). The analysis outputs the features of the input data, including the sample size, marker density, linkage disequilibrium (LD) decay, heritability (h2), principal components (PCs), and accuracy of prediction. These features are combined with existing database for cluster analysis to identify the nearest neighbor (NN) to the input data. If gBLUP is the best method for the NN, the calculation is complete. Otherwise the best method for the NN is selected for the new data and the prediction accuracy is added to the database for cluster analysis. The process is iterated until the best method is identified for the NN, or the number of iterations is above a threshold which is three by default.

**
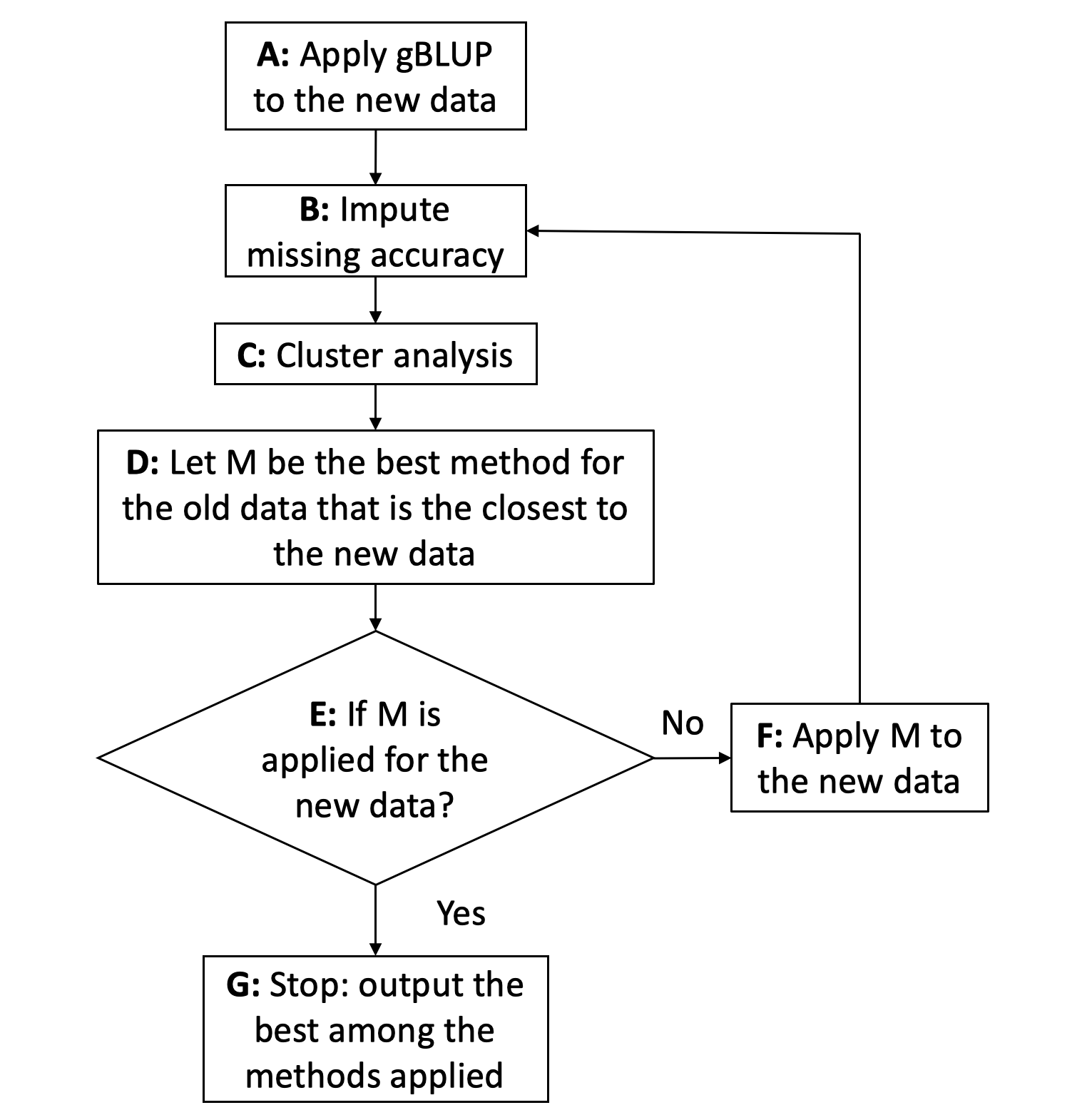
**

**Figure S2. Diagram of interactive and dynamic evaluation to find the optimum prediction method for a new dataset.**For a new dataset, gBLUP is applied to get prediction accuracy using fivefold cross-validation with ten iterations (A). The properties of new data are added to the knowledge database, which contains the prediction accuracies for all implemented prediction methods and properties of datasets examined, including heritabilities. The missing prediction accuracies are imputed with the nearest neighbor algorithm (B). All features are used for cluster analysis using the shortest distance algorithm (C). For the old dataset that is closest to the new data, search the best prediction method for the old dataset (D). If the best method is not applied to the new data yet, apply the method to the new data (E and F) and go back to impute prediction accuracies—otherwise, iteration stop for output. After getting results to users, MMAP compute all the missing prediction accuracies for the new data to update knowledge database when computing resources are available.

**
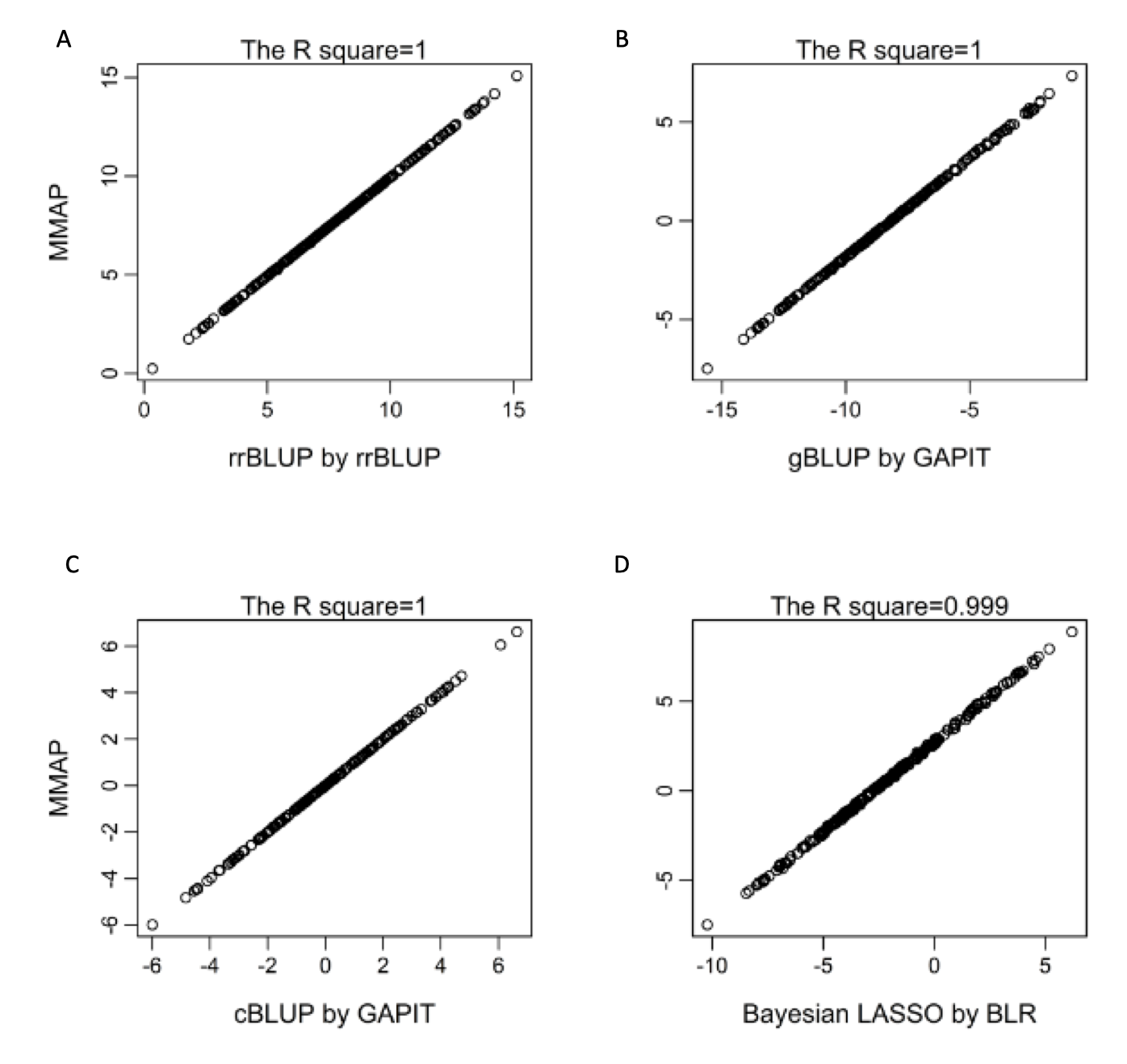
**

**Figure S3. Identical estimates of genomic breeding values using MMAP and other packages.**

The genomic breeding values were estimated using Ridge Regression, genomic Best Linear Unbiased Prediction (gBLUP), compressed BLUP (cBLUP), and Bayesian LASSO. The estimates using MMAP are compared with the estimates using rrBLUP for ridge regression, BLR for Bayesian LASSO, and GAPIT for gBLUP and cBLUP. MMAP uses the same random seeds for Bayesian LASSO so they generate the identical results. Other methods do not involve random sampling.

**
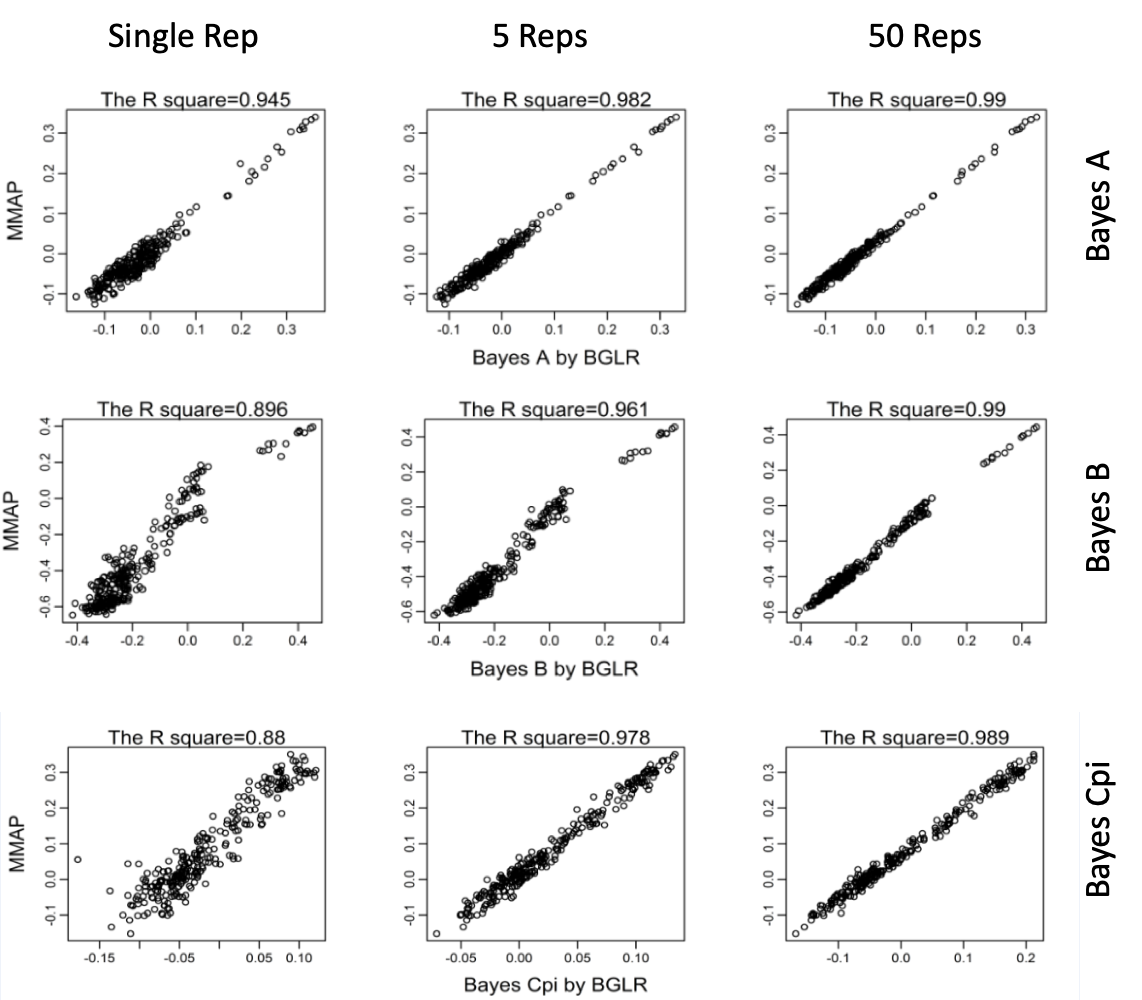
**

**Figure S4. Comparison between estimated breeding values using multiple methods involved random sampling.** Breeding values were estimated by MMAP and BGLR packages for three Bayesian methods (A, B, and Cpi). Because random sampling is used for these methods, there are variation between and within software packages. The comparisons between software packaged were conducted on a single replicate (Rep) and averages over five and 50 replicates. The more replicates, the more similar between the two software packages.

**
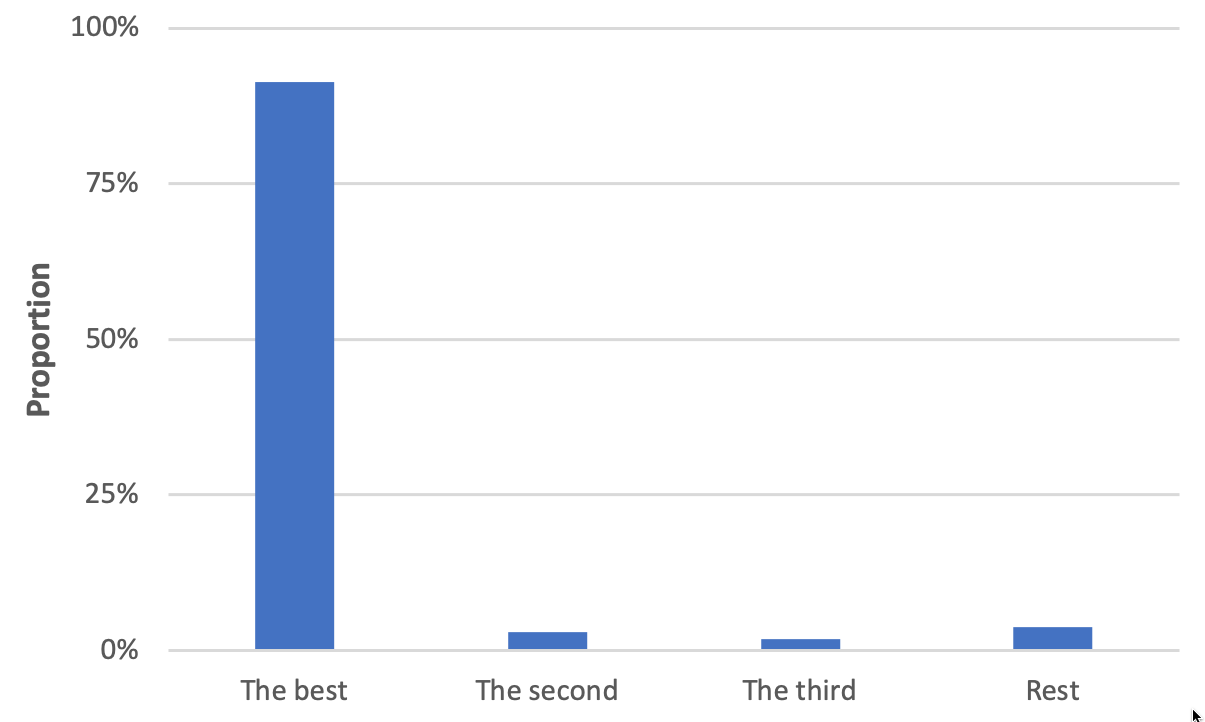
**

**Figure S5. Performances of MMAP for find the best or near best prediction methods.** The performances were measured on 269 traits across 11 species. The average number of runs was 2.93 with standard deviation of 0.75. The proportions of finding the best, the second and the third best prediction methods were 91.32%, 3.03%, and 1.80%. The proportion of missing the first top three prediction methods was below 4%.


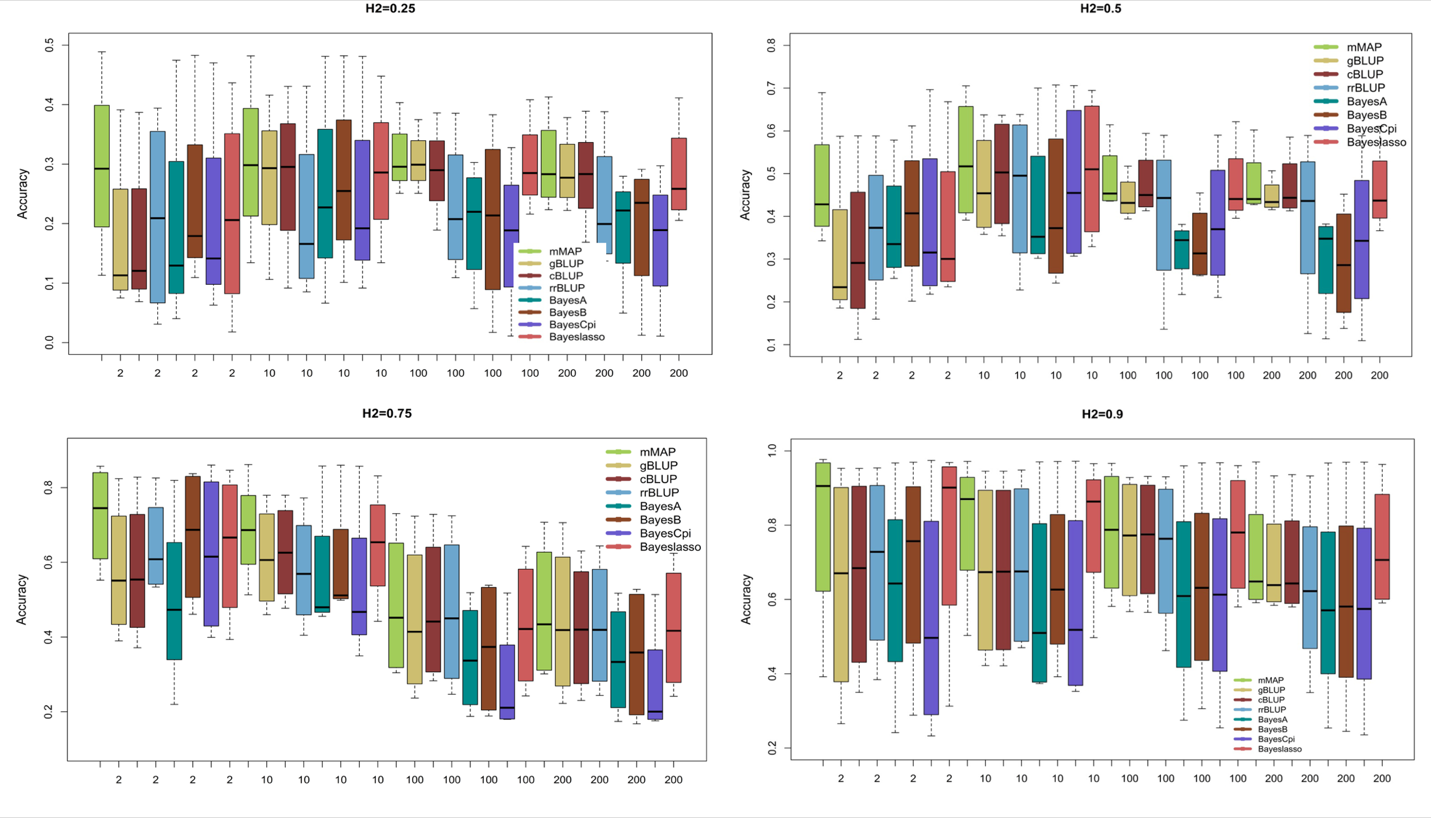


**Figure S6. Distribution of prediction accuracy using different prediction methods.** The methods include MMAP, Bayes A, Bayes B, Bayes Cpi, Bayesian LASSO, the genomic Best Linear Unbiased Prediction (gBLUP), compress BLUP (cBLUP), and ridge regression (rrBLUP). The distribution of prediction accuracies were derived from simulated phenotypes with 100 replicates. The phenotypes were controle by 2, 10, 100, 0r 200 genes with heritability of 0.25, 0.5, 0.75, and 0.9. The genes were sampled from 55,000 SNPs genotyped on 282 maize inbred lines.


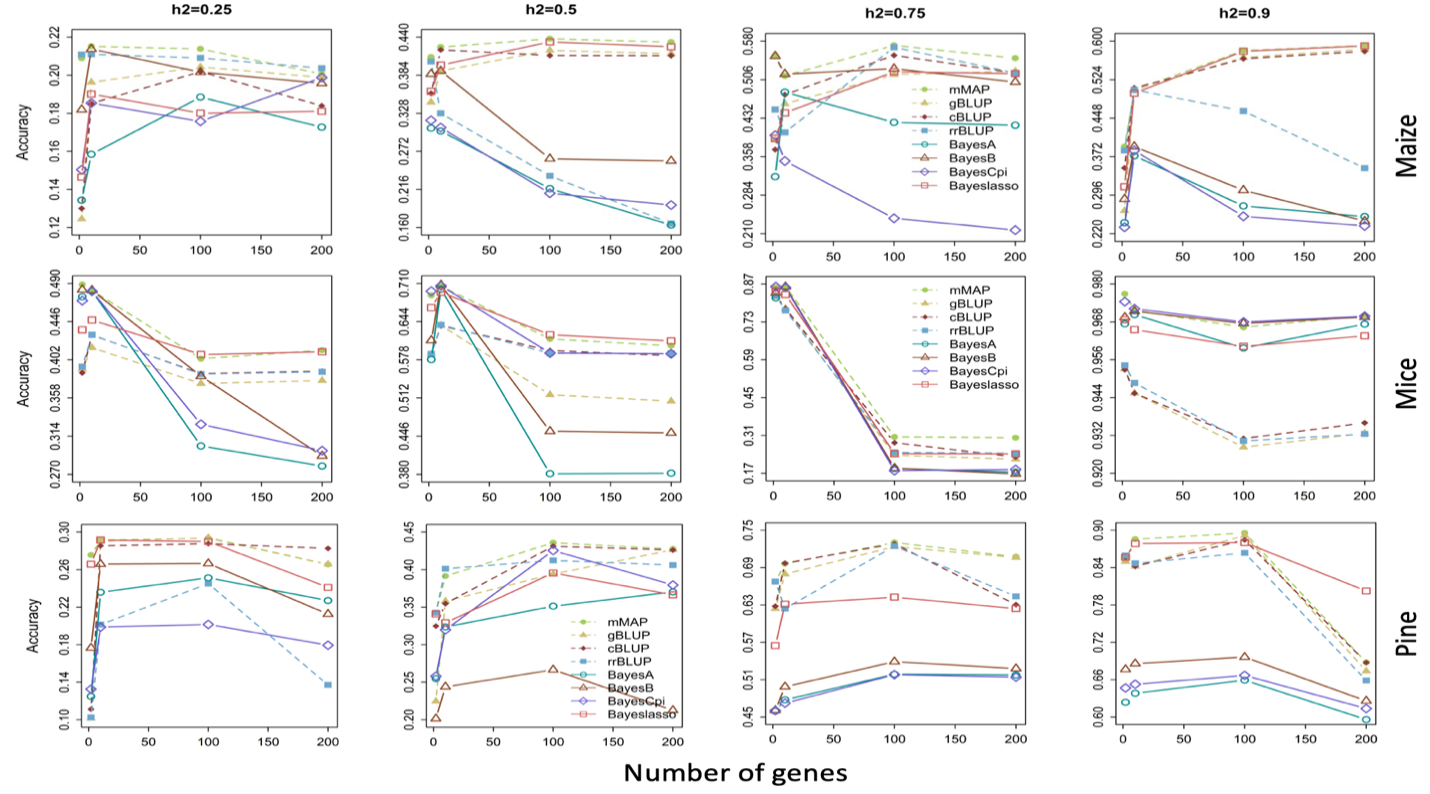


**Figure S7. Average of prediction accuracy using different prediction methods.** The methods include MMAP, Bayes A, Bayes B, Bayes Cpi, Bayesian LASSO, the genomic Best Linear Unbiased Prediction (gBLUP), compress BLUP (cBLUP), and ridge regression (rrBLUP). The distribution of prediction accuracies were derived from simulated phenotypes with 100 replicates. The phenotypes were controle by 2, 10, 100, 0r 200 genes with heritability of 0.25, 0.5, 0.75, and 0.9. The genes were sampled from 55,000 SNPs genotyped on 282 maize inbred lines.

**Table S1. Prediction accuracy using different prediction methods*.**

| **Traits** | **mMAP** | **gBLUP** | **cBLUP** | **rrBLUP** | **BayesA** | **BayesB** | **BayesCpi** | **Bayeslasso** |
| --- | --- | --- | --- | --- | --- | --- | --- | --- |
| EarHT | 0.54 | 0.51 | 0.54 | 0.51 | 0.45 | 0.51 | 0.47 | 0.51 |
| DTS | 0.88 | 0.87 | 0.88 | 0.87 | 0.69 | 0.80 | 0.72 | 0.87 |
| DTA | 0.89 | 0.89 | 0.88 | 0.88 | 0.75 | 0.83 | 0.84 | 0.88 |
| ASI | 0.42 | 0.33 | 0.34 | 0.28 | 0.27 | 0.37 | 0.33 | 0.39 |
| PH | 0.54 | 0.53 | 0.47 | 0.35 | 0.49 | 0.53 | 0.44 | 0.50 |
| FLL | 0.62 | 0.60 | 0.56 | 0.58 | 0.52 | 0.60 | 0.53 | 0.61 |
| FLW | 0.63 | 0.59 | 0.53 | 0.59 | 0.59 | 0.60 | 0.62 | 0.61 |
| SLL | 0.60 | 0.59 | 0.58 | 0.55 | 0.57 | 0.58 | 0.61 | 0.59 |
| SLW | 0.39 | 0.34 | 0.33 | 0.37 | 0.27 | 0.35 | 0.34 | 0.35 |
| FLDW | 0.55 | 0.53 | 0.52 | 0.53 | 0.32 | 0.48 | 0.42 | 0.54 |
| SLDW | 0.66 | 0.63 | 0.66 | 0.65 | 0.51 | 0.59 | 0.58 | 0.63 |
| HTLC | 0.43 | 0.43 | 0.39 | 0.43 | 0.38 | 0.43 | 0.38 | 0.43 |
| BA | 0.50 | 0.49 | 0.47 | 0.49 | 0.45 | 0.49 | 0.46 | 0.49 |
| BD | 0.26 | 0.25 | 0.24 | 0.25 | 0.22 | 0.26 | 0.25 | 0.26 |
| BLC | 0.47 | 0.47 | 0.44 | 0.46 | 0.37 | 0.42 | 0.43 | 0.46 |
| CWAC | 0.46 | 0.44 | 0.43 | 0.45 | 0.30 | 0.41 | 0.36 | 0.44 |
| CWAL | 0.36 | 0.35 | 0.34 | 0.36 | 0.30 | 0.29 | 0.21 | 0.36 |
| Plantheight | 0.56 | 0.56 | 0.54 | 0.56 | 0.32 | 0.40 | 0.39 | 0.36 |
| Earheight | 0.56 | 0.56 | 0.56 | 0.55 | 0.36 | 0.42 | 0.36 | 0.39 |

***** The methods include MMAP, Bayes A, Bayes B, Bayes Cpi, Bayesian LASSO, the genomic Best Linear Unbiased Prediction (gBLUP), compress BLUP (cBLUP), and ridge regression (rrBLUP).

**Table S2. Average of prediction accuracy using different prediction methods*.**

| **Simulation traits**  **(data, h^2^, and NQTN)** | **MMAP** | **gBLUP** | **cBLUP** | **rrBLUP** | **BayesA** | **BayesB** | **Bayes**  **Cpi** | **Bayes LASSO** |
| --- | --- | --- | --- | --- | --- | --- | --- | --- |
| 55Kmaize1_0.25,2 | 0.21 | 0.12 | 0.13 | 0.21 | 0.13 | 0.18 | 0.15 | 0.15 |
| 55Kmaize1_0.25,10 | 0.22 | 0.20 | 0.19 | 0.21 | 0.16 | 0.21 | 0.19 | 0.19 |
| 55Kmaize1_0.25,100 | 0.21 | 0.20 | 0.20 | 0.21 | 0.19 | 0.20 | 0.18 | 0.18 |
| 55Kmaize1_0.25_200 | 0.20 | 0.20 | 0.18 | 0.20 | 0.17 | 0.20 | 0.20 | 0.18 |
| 55Kmaize1_0.75,2 | 0.55 | 0.39 | 0.37 | 0.45 | 0.32 | 0.55 | 0.40 | 0.39 |
| 55Kmaize1_0.75,10 | 0.51 | 0.46 | 0.48 | 0.40 | 0.48 | 0.52 | 0.35 | 0.44 |
| 55Kmaize1_0.75,100 | 0.57 | 0.52 | 0.55 | 0.57 | 0.42 | 0.53 | 0.24 | 0.52 |
| 55Kmaize1_0.75_200 | 0.55 | 0.52 | 0.52 | 0.52 | 0.42 | 0.50 | 0.22 | 0.52 |
| soybean_0.5,2 | 0.45 | 0.19 | 0.11 | 0.16 | 0.36 | 0.45 | 0.37 | 0.24 |
| soybean_0.5,10 | 0.61 | 0.52 | 0.59 | 0.59 | 0.38 | 0.45 | 0.59 | 0.62 |
| soybean_0.5,100 | 0.47 | 0.44 | 0.47 | 0.47 | 0.34 | 0.36 | 0.31 | 0.45 |
| soybean_0.5,200 | 0.45 | 0.44 | 0.46 | 0.47 | 0.33 | 0.36 | 0.31 | 0.45 |
| Pine_0.9,2 | 0.85 | 0.85 | 0.86 | 0.86 | 0.62 | 0.68 | 0.65 | 0.86 |
| Pine_0.9,10 | 0.89 | 0.84 | 0.84 | 0.85 | 0.64 | 0.69 | 0.65 | 0.88 |
| Pine_0.9,100 | 0.90 | 0.89 | 0.88 | 0.86 | 0.66 | 0.70 | 0.67 | 0.88 |
| Pine_0.9,200 | 0.69 | 0.67 | 0.69 | 0.66 | 0.60 | 0.63 | 0.61 | 0.80 |

* The methods include MMAP, Bayes A, Bayes B, Bayes Cpi, Bayesian LASSO, the genomic Best Linear Unbiased Prediction (gBLUP), compress BLUP (cBLUP), and ridge regression (rrBLUP). The distribution of prediction accuracies were derived from simulated phenotypes with 100 replicates. The phenotypes were controled by 2, 10, 100, 0r 200 genes with heritability of 0.25, 0.5, 0.75, and 0.9. The genes were sampled from 55,000 SNPs genotyped on 282 maize inbred lines, 6,704 SNPs from 269 soybean inbred lines, and 48,530 SNPs from 927 pines. Only one heritability level was conducted for soybean (0.5) and pines (0.9)
